# A Review on Cementitious Self-Healing and the Potential of Phase-Field Methods for Modeling Crack-Closing and Fracture Recovery

**DOI:** 10.3390/ma13225265

**Published:** 2020-11-21

**Authors:** Sha Yang, Fadi Aldakheel, Antonio Caggiano, Peter Wriggers, Eddie Koenders

**Affiliations:** 1 Institute of Construction and Building Materials, Technical University of Darmstadt, Franziska-Braun-Straße 3, 64287 Darmstadt, Germany; yang@wib.tu-darmstadt.de (S.Y.); koenders@wib.tu-darmstadt.de (E.K.); 2 Institute of Continuum Mechanics, Leibniz Universitaet Hannover, An der Universitaet 1, 30823 Garbsen, Germany; wriggers@ikm.uni-hannover.de; 3 CONICET and LMNI-FIUBA, Universidad de Buenos Aires, Buenos Aires C1127AAR, Argentina

**Keywords:** self-healing, phase-field, cement-based systems, precipitation, reaction, fracture, transport

## Abstract

Improving the durability and sustainability of concrete structures has been driving the enormous number of research papers on self-healing mechanisms that have been published in the past decades. The vast developments of computer science significantly contributed to this and enhanced the various possibilities numerical simulations can offer to predict the entire service life, with emphasis on crack development and cementitious self-healing. The aim of this paper is to review the currently available literature on numerical methods for cementitious self-healing and fracture development using Phase-Field (PF) methods. The PF method is a computational method that has been frequently used for modeling and predicting the evolution of meso- and microstructural morphology of cementitious materials. It uses a set of conservative and non-conservative field variables to describe the phase evolutions. Unlike traditional sharp interface models, these field variables are continuous in the interfacial region, which is typical for PF methods. The present study first summarizes the various principles of self-healing mechanisms for cementitious materials, followed by the application of PF methods for simulating microscopic phase transformations. Then, a review on the various PF approaches for precipitation reaction and fracture mechanisms is reported, where the final section addresses potential key issues that may be considered in future developments of self-healing models. This also includes unified, combined and coupled multi-field models, which allow a comprehensive simulation of self-healing processes in cementitious materials.

## 1. Introduction

Concrete is characterized by its high compressive strength, a wide availability of its raw materials, and simple production methods, which is the main reason that it became the most commonly used construction material in the world [1,2]. However, its low tensile strength is the main reason that various types of cracks can occur in a concrete element that may adversely affect its service life [3]. While under internal, external, or environmental load, open or closed micro- and/or meso cracks may develop inside a concrete element that may successively result in a loss of structural integrity [4]. Open surface cracks may also allow water or hazardous substances to enter and thereby severely impairing its durability [5,6]. Therefore, improving the durability of concrete structures, asks for a limitation or reduction of the number of cracks where self-healing strategies could be solution. In the last decades, enormous efforts have already been done to develop various kinds of self-healing methods for cementitious systems [7,8,9,10,11,12,13,14,15]. Most comprehensive scientific report so far is the RILEM TC-221-SHC [16], that summarizes the current research progress and defines the difference between “autogenic” and “autonomic” self-healing methods, depending on whether crack closure happens due to either the material itself [17,18,19,20], or is triggered by means of engineered additions [13,14,21,22,23,24,25,26,27,28,29,30].

From a modeling point of view, the presently existing numerical approaches can be grouped according to the nature of their particular self-healing mechanism into (1) chemical reaction-based models [12,31,32,33,34], for predicting carbonation, hydration, polymerization and precipitation phenomena; (2) transport phenomena-based models [35,36], in which the phases affecting the healing processes are transported through the concrete pore-structure network; and (3) fracture-based models, smeared [37,38,39,40,41,42,43,44,45,46] and discrete [47,48,49,50,51,52,53] crack approaches for predicting strength recoveries of self-healing systems.

When considering the number and type of experiments required to study the performance of self-healing concrete, it turns out that optimizing self-healing mechanisms through extensive experimental studies is a very demanding task. However, this task becomes more doable when employing numerical simulation models. However, most existing models do not incorporate physically/chemically driven boundary movements for an accurate simulation of solid–liquid interfaces. To overcome these difficulties, phase-field (PF) methods have been proposed as a powerful tool for handling moving interfaces caused by phase transitions [54,55,56]. In conventional numerical models for phase transformations and microstructural evolutions, interfaces are considered to be infinitely sharp and have to be schematized explicitly [57,58,59]. It leads to incompatibilities that makes calculations very complex and difficult to implement in a computer program. Contrarily, PF methods are based on thermodynamic principles and assume a diffuse interface, which makes them suitable for solving complex morphological evolutionary processes. The evolution of the “field”, over time and space, is controlled by the nonlinear Cahn–Hilliard diffusion equation and its relaxation by the Allen–Cahn equation [60,61]. For concrete, a self-healing mechanism is physically almost similar to a dissolution and/or precipitation principle that evolves at the cracked surfaces. It makes a PF modeling approach very suitable for solving this type of moving interface problems at cracked surfaces, caused by phase transformations.

This article provides a review on existing models to simulate self-healing in cracked concrete, with emphasis on PF methods. After the introduction in Section 1, the currently available self-healing methods for concrete are reported in Section 2. In Section 3, the possibility of using PF methods for simulating self-healing in concrete is presented and discussed. Then, in Section 4, the basic equations of a PF method are presented. Next, in Section 5 and Section 6 existing PF techniques for precipitation and fracture in concrete are reported, respectively. Finally, items that should be addressed in self-healing models along with future research priorities and a concluding discussion on the whole article is given in Section 7.

## 2. Self-Healing Mechanisms in Concrete

In general, self-healing processes in cement-based materials can be divided into two categories: (1) autogenous self-healing and (2) autonomous self-healing [9,62,63]. Autogenous self-healing involves only the original components of a concrete. These components may, due to their specific chemical compositions, promote crack healing under favorable environmental conditions, driven by chemical reactions or transitions [10,64,65]. However, autonomous self-healing processes can only take place with the help of healing additives, such as microcapsules that may contain healing agents like polymers or bacterial spores [14,66]. Autogenous healing mechanisms have a limited healing capacity, typically only being able to heal cracks of about 100–150 μm in width [67]. In contrast to this, autonomous mechanisms can easily heal cracks up to 300 μm and sometimes even more than 1 mm [67]. These self-healing mechanisms are described below in detail.

### 2.1. Autogenous Self-Healing

**Figure 1:**
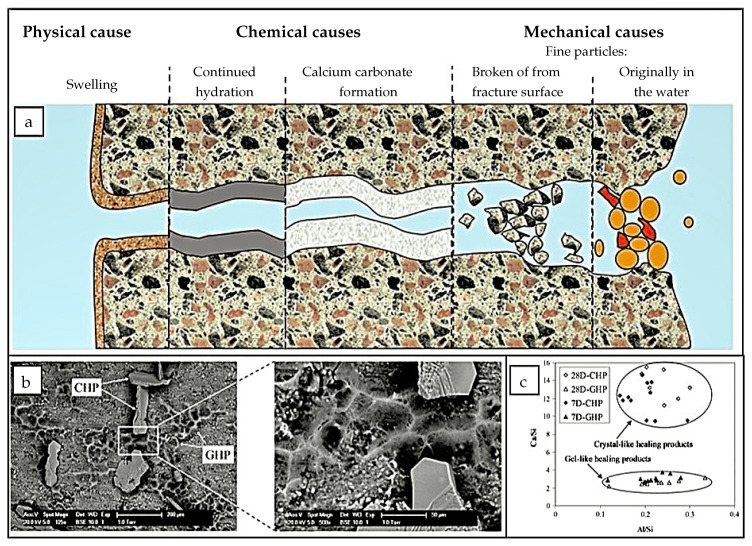
The autogenous self-healing mechanisms, products, and their corresponding chemical composition. (**a**) Schematic representation of the mechanisms of autogenic self-healing. Reproduced with permission from the authors of [70]. Copyright 2013, Springer. (**b**) Morphology of healing products (GHP refers to the gel-like healing product and CHP refers to the crystal-like healing product). Reproduced with permission from the authors [76]. Copyright 2013, Elsevier. (**c**) Ratios of Ca/Si and Al/Si of healing products with time. Reproduced with permission from the authors of [76]. Copyright 2013, Elsevier.

Autogenous self-healing has been extensively investigated in the last decades [9,10,65,68,69], mainly by using experimental techniques. Figure 1a shows three main categories: physical, chemical, and mechanical healing. The physical healing mechanism is the process where the crack surface inside a cement matrix absorbs water and causes volume expansion [70,71]. The chemical healing mechanism consists of two main reactions, namely, a further hydration of the still unhydrated cement clinker inside a concrete, generating additional Calcium Silicate Hydrates (C-S-Hs), and carbonation of the additionally formed portlandite [65,72,73,74]. Finally, mechanical healing mechanisms refers to the filling of a crack with fine cement particles, which appear in a crack by water transport or diffusion [72]. The chemical mechanism is the primary and most promising healing method for hardened concrete at a young age [16]. Due to the relatively high content of unhydrated cement particles in these concretes, continuing hydration will still be possible and may result in a healing of cracks [18,64]. At later ages after crack initiation, the formation and growth of calcium carbonate crystals (CaCO_3_) becomes the main healing mechanism [75]. Figure 1b,c shows the main healing products and their chemical components.

**Figure 2:**
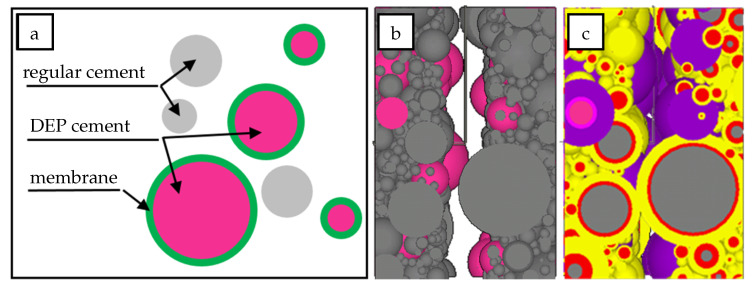
Self-healing with Dissoluble Encapsulated Particles (DEP): (**a**) Schematic representation of regular cement blended DEP cement [12]. (**b**) Initial state of microstructure by vol.−10% cement replacement by DEP [12]. (**c**) A high pH value will cause the DEP capsule to rupture, the healing agent will be released and a special hydration reaction with accompanying volume expansion will begin [77].

To improve the effectiveness of autogenous crack repair, an improved self-healing method called Dissoluble Encapsulated Particles (DEP) has been proposed [11,12,77]. In this self-healing method a certain amount of cement in a concrete mixture remains unhydrated for a predefined period of time because of the pre-encapsulation of certain cement fractions which are covered with a thin membrane that can dissolve whenever it is affected by a crack (Figure 2). A crack in a cementitious surface may open the DEP membrane due to either (1) a dissolution mechanism caused by low pH-conditions, i.e., due to increased CO_2_ ingress, or (2) by mechanical fracture. After this happened, the original unhydrated cement will be exposed to the local environmental temperature and humidity conditions causing the cement to react and finally close the crack [77].

### 2.2. Autonomous Self-Healing

Autonomous self-healing is a method to improve the effectiveness of self-healing mechanisms for concrete, by either embedding encapsulated or non-encapsulated additions [62,67]. Until now, addition of encapsulated agents (micro/meso < 1 mm, macro ≥ 1 mm) is the most preferred method adopted for autonomous self-healing concrete [67], which may contain mineral [78,79], bacteria [14,28,80,81,82,83,84,85,86,87,88], and polymers [15,89,90]. Non-encapsulated additions may also contain these listed substances, but are added to a mixture in a pure, non-encapsulated, form where they become active directly after mixing of the concrete [91,92,93].

#### 2.2.1. Self-Healing Based on Mineral Admixtures

**Figure 3:**
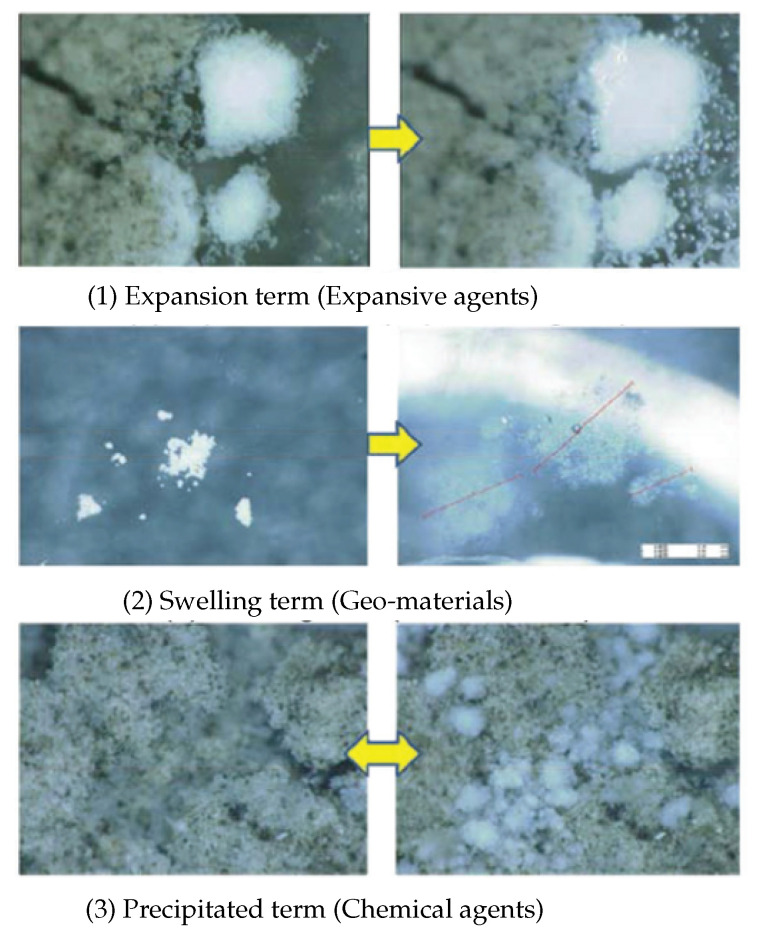
Three main self-healing mechanisms using mineral admixtures. Reproduced with permission from the authors of [79]. Copyright 2010, JCI.

Mineral admixtures are materials that are mixed in a concrete and react with water to form reaction products with an expanded volume to heal cracks developed in an already hardened concrete. With this healing mechanism [13,91,94,95], crack widths up to 120 μm can be repaired [67]. Depending on the type of mineral additives, three subcategories can be identified: (1) expansive additives, (2) geo-material based additives, and (3) chemical agents (crystalline additives) (Figure 3). Expansive additives develop reaction products with an increased volume that can fill the cracks [96]. Commonly used are sulfoaluminate based expansive additives (C-S-A) [78]. The geo-material-based additives consist of silicon dioxide, sodium aluminum silicate hydroxide, and bentonite clay, which have the capacity to swell [79,97,98]. When this type of geo-material is exposed to water, its volume may increase 15–18 times its initial dry volume [79]. The most basic crystalline additive is tricalcium silicate (C_3_S), which is the main clinker component in cement and reacts with water to form calcium silicate hydrate C-S-H phases [26].

#### 2.2.2. Self-Healing Based on Bacteria

**Figure 4:**
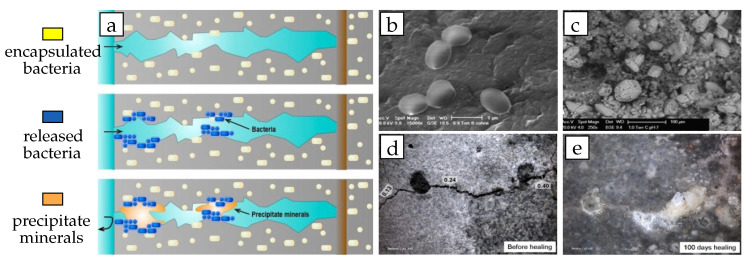
Self-healing mechanism using bacterial spores. (**a**) Schematic diagram of bacterial repair of concrete cracks. Bacteria on the surface of the crack are activated by water and precipitate minerals such as calcite to seal the crack and protect the reinforcement from external chemical attack. Reproduced with permission from the authors of [99]. Copyright 2018, Elsevier. (**b**) ESEM photomicrograph (15,000× magnification) of *B. cohnii* spores, showing that spore diameter sizes are up to 1 μm. Reproduced with permission from the authors of [80]. Copyright 2010, Elsevier. (**c**) Mineral precipitates (20–80 μm sized) on crack surfaces (250× magnification). Reproduced with permission from the authors of [80]. Copyright 2010, Elsevier. (**d**) Stereomicroscopic images of crack-healing process in bio-chemical agent-based specimen before and (**e**) after 100 days healing. Reproduced with permission from the authors of [81]. Copyright 2011, Elsevier.

A certain category of bacteria can be applied for healing cracks in concrete [28]. It results in a closed crack which is watertight and has a limited capacity to restore the mechanical strength of a concrete [80,81,82]. The maximum crack width that can be healed with this system are ~150 μm [83], which is rather limited whenever compared with other healing systems. Figure 4 shows a schematic impression of a fractured concrete with microencapsulated bacterial spores and the results of previous experiments [80,81,99].

Bacteria provide an important reaction component in a self-healing mechanism, where they are enhancing the calcium carbonate CaCO_3_ production, needed for crack closing [100]. During healing, the mechanism passes the following two sequential steps; (1) conversion of calcium lactate and (2) hydrolysis of urea through (ureolytic) bacterial metabolism. In the first mechanism, oxygen and water penetrate into the concrete interior through cracks where the bacteria are activated to convert calcium lactate into CaCO_3_ crystals and CO_2_. Portlandite particles near the cracks will further react with CO_2_ to produce more CaCO_3_ which precipitates at the crack surfaces [81]. In the second mechanism, many components capable of producing organic urea (e.g., *Bacillus cohnii*, *Sphaericus*, *Subtilis*, *Pasteurii*, *Megaterium*, and *Sporosarcina ureae*) can act as a catalyst during the self-healing process [101]. As it undergoes demineralization, negatively charged bacterial cells take up components from the cell wall and then react to CaCO_3_ precipitates [102].

The efficiency of the precipitates generated by bacterial induction is determined first by the available water content and moisture movement in the concrete matrix [103,104], and second by the concentration of calcium ions, the pH of the pore solution, the concentration of inorganic carbon and by the presence of nucleation sites [105,106]. The first three are available in the concrete matrix, while the last one is related to the type of bacteria used [82]. In addition, factors that affect the effectiveness of healing include (1) the type of carrier (direct [107], encapsulated [108] containers like clay and aggregates [109,110]) and (2) the concrete compatible chemical reactions taking place in producing CaCO_3_ [86,111].

#### 2.2.3. Self-Healing Based on Adhesive Agents

**Figure 5:**
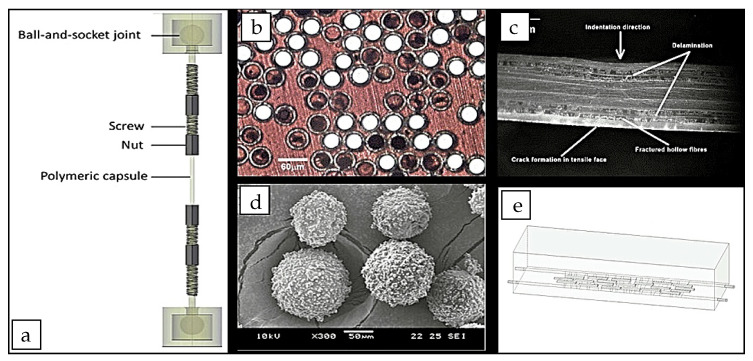
Self-healing mechanism based on adhesive agents. (**a**) Test setup used to determine the tensile strength of laboratory-scale hollow glass tubes with an outer hole of diameter 5 mm and an inner pin of diameter 3 mm. Reproduced with permission from the authors of [117]. Copyright 2015, Elsevier. (**b**) Hollow glass fibres of 60 μm external diameter with a hollowness of 50%. (**c**) Cross section through impact damaged hybrid solid glass/hollow glass/epoxy laminate. Reproduced with permission from the authors of [120]. Copyright 2005, Elsevier. (**d**) Spherical microcapsules with diameter of 120 ± 33 μm. Reproduced with permission from the authors of [122]. Copyright 2012, Elsevier. (**e**) Short glass/ceramic capsules attached to reinforcement, Reproduced with permission from the authors of [118]. Copyright 2015, Elsevier.

This method is based on injecting adhesives into a crack to induce manual healing [112,113]. The crack widths which can be healed with these systems vary from 50 μm up to 250–300 μm [67,114]. Adhesive agents can be divided into one-component and multicomponent systems. Commonly used one-component adhesive agents are polyurethane [115] and epoxy [116]. Multicomponent adhesives are methylmethacrylate [117] and ureaformaldehyde/epoxy [113]. Adhesive agents are encapsulated in spherical capsules [112], tubular-shaped capsules [117,118], and hollow fibers [119,120,121] that are mixed with fresh concrete (Figure 5). When cracks occur, rupture of the encapsulation takes place, where the adhesive will be released into the crack by capillary action, initiating crack healing with time.

## 3. Phase-Field Methods for Modeling Concrete Self-Healing

The Phase Field (PF) method for simulating lower scale micro- and/or mesostructural cracking in materials has got an enormous upswing in the last decades. However, so far classical PF applications were focusing on the distribution of non-reactive multi-phase systems [123]; solidification problems [55,124]; solid-state phase changes [125,126]; grain growth, nucleation, and coalescence processes [127,128,129]; dislocation dynamics [130]; temperature inducing phase transformations [131]; liquid-phase sintering [132]; mass transport phenomena [133]; hydrodynamics [134]; and electromigration [135]. Recently, many problems in solid mechanics deal with the use of PF for describing fracture phenomena and to capture complex crack patterns [136,137,138,139,140]. Based on the present literature review, the following can be summarized.

- PF is an extremely powerful mathematical modeling scheme for accurately describing physical movements of phase boundaries.
- PF was mainly employed for solving solidification dynamics, material phase changes/separations, growing phases driven by chemo-kinetics and transport phenomena, nucleation and coalescence processes between particles in micro-to-mesostructures.
- PF has been successfully employed in fracture mechanics to capture the cracking response of brittle/ductile materials without the need for employing Discrete Crack Approaches (DCAs) and/or Smeared Crack Approaches (SCA).

Because of this, and as also supported by various state-of-the-art reports [55,56,141,142,143,144], PF models can be employed for self-healing of brittle or plastic (ductile) materials in a fundamental and consistent way. It will combine the impact of two main phase changes that occur simultaneously in a self-healing mechanism, i.e., chemical reactions and fracture. Gradual changes from the fully-cracked (failure) to the uncracked configuration can be driven through the so-called Phase-Field order parameter (ϕ). It will provide a smooth transition of all relevant phenomena between the fully cracked configuration and the intact material phases: this strength and crack recoveries actually represent the self-healing process. The governing equations of the proposed unified model will be derived in the framework of thermodynamics concepts, in terms of kinematics and balance equations, dissipation inequality and constitutive laws. Particularly, the free energy will be considered as the sum of the contributions due to elasticity, reaction PF and fracture PF. The free energy of the system is described in a unified form over the entire phase transition region. In this regard, the advantage of the PF method over other competitive numerical methods is its enormous capability of capturing movements of interfaces, without the need for introducing any additional ad hoc technique, criteria and/or remeshing strategies, and also without any explicit tracking of the actual interface positions of these coupled processes. The governing equations of PF models for chemical/moisture reactions and fracture processes, associated with self-healing, as well as the coupling among them, can be formulated in a unified PF framework. The next sections report a review on the available formulations for a unified and coupled set of PF approaches for modeling reactions and fracture of self-healing mechanisms in concrete.

## 4. Main Equations of a Phase-Field Approach

**Figure 6:**
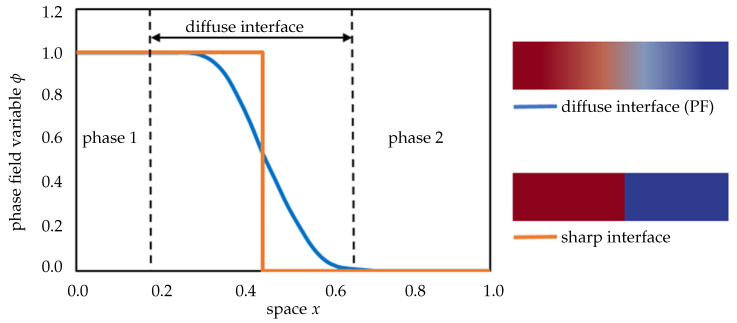
Schematic representation of sharp interface model and phase-field model.

The phase-field (PF) approach is a very powerful technique to simulate complex physical phenomena in multi-field environments. The main attributions of this approach are simplicity and generality. A popular PF application is a diffusion interface model that is frequently used to simulate phase transformation problems in materials research [145,146,147]. The classical PF method is formulated based on the theory of Ginzburg and Landau, elaborated in the 1950s [148]. Compared with the sharp interface model, the PF diffusion interface model has the important advantage that no boundary conditions are specified on the interface between the different domains (Figure 6). A diffusive order parameter ϕ is a continuous function coordinate of time and space, which indicates each phase to convert between 0∼1 or −1∼1 within a thin translation layer [54,144]. Moreover, ϕ is controlled by a set of coupled partial differential equations that can be discretized and solved numerically by evolving the equations. Any phase transformation is driven by a reduction of the free energy of the system *F*, which can be described by a set of conserved $c_{i}$ and non-conserved $\phi_{i}$ field variables. The domain of the model is the entire phase transition system. The free energy of the system consists of the energy contributions from the homogenous bulk phases $F_{bulk}$ and the diffuse interface region $F_{int}$, according to [146]

(1) $$F\left(\phi ,c\right)=F_{bulk}+F_{int}=\int_{V}\left[f_{loc}\left(\phi ,c\right)+f_{int}\left(\nabla \phi ,\nabla c\right)\right]\;dV$$

where $f_{loc}$ defines the local free energy density (including chemical, interfacial and elastic strain free energy density), while $f_{int}$ defines the diffusive interface energy density.

From the computational point of view, monolithic or staggered algorithms can be computed to solve the problem unknowns, in which mechanical, chemical, interface, and phase-field variables are computed simultaneously or sequentially, respectively. For more details the interested reader is referred to the works in [136,149,150]. In those works, robust and efficient monolithic schemes were employed for the numerical implementation.

### 4.1. Evolution Equation

The generalized PF method is represented by the Ginzburg–Landau or Onsager kinetic equation combined with the well fitted Landau– or Redlich–Kister-type free energy density functionals, which are dependent on both conserved and non-conserved field variables [146]. The time-dependent evolution of the conserved field variables (chemical concentration) is defined using a modified Cahn–Hilliard equation [151], while the Allen–Cahn equation describes the transformations with non-conserved variables (e.g., crystal orientation, long-range order, crystal structure, and elastic strain) [152].

The Cahn-Hilliard equation is

(2) $$\frac{\partial c_{i}\left(r,t\right)}{\partial t}=\nabla \cdot M_{c}\nabla \frac{\delta F}{\delta c_{i}\left(r,t\right)}$$

where $c_{i}$ is the conserved concentration field variable, $M_{c}$ is the kinetic coefficient of diffusion (associated mobility), *t* is the time and *r* is the spatial coordinate, ∇ is a vector of partial derivative operator, and δ denotes the variational derivation of the functional *F*.

The Allen–Cahn equation is

(3) $$\frac{\partial \phi_{i}\left(r,t\right)}{\partial t}=-L_{\phi }\frac{\delta F}{\delta \phi_{i}\left(r,t\right)}$$

where $\phi_{i}\left(r,t\right)$ are the *i* different structure field variables with *i* = 1, 2 ..., *n*, while $L_{\phi }$ is the kinetic structure operators (order parameter mobility). Depending on the problem, $L_{\phi }$ has different expressions [124,153,154].

### 4.2. Local Free Energy Function

The local free energy function is a key component in the PF model [155]. This function describes the free energy density of each bulk phase, whose coefficients are obtained from thermodynamic data [153]. The expression of the local free energy depends on the problem of interest. For example, a double-well form is often used for solidification [147,156]. When dealing with an electromigration problem, a double-obstacle potential is usually applied [55,157]. A crystalline energy function is used to describe an overlapped dislocation of an elastically anisotropic crystal [158,159,160]. When the problem is temperature-controlled, as in the melting and solidification processes of crystals, the local free energy function contains a temperature field [161,162]. In such cases, the phase-field is needed to be coupled with a temperature field [161,162,163,164,165]. Furthermore, a Landau-type polynomial potential can be applied for the treatment of a solid-state phase transformation [166,167,168,169,170,171]. Table 1 summarizes examples of the universal expressions, the graphs of the local free energies and existing phase-field applications.

## 5. Phase-Field Modeling of Precipitation Reaction Mechanisms

Self-healing of concrete can be numerically treated as a precipitation process of solutes at the solid–liquid crack interface [215,216], which is time-dependent and controlled by chemical reactions and diffusion [36,217]. When the rate of the chemical reactions at the interface is sufficiently high and there is no fluid flow, diffusion will be the only mechanism left for solute transport. The whole process is then a diffusion-controlled precipitation one [216]. However, when the chemical kinetics is slow enough, the precipitation process becomes chemically determined [218]. A review of existing models for self-healing that are based on chemical reactions show that these models are employing a reaction-diffusion process to describe the self-healing evolution [12,31,32,33,34]. These models focus on two processes: (1) the diffusion mechanism where dissolved ions (e.g., calcium ions) are transferred from the concrete interior toward the surface of the crack, and (2) the precipitation of mineral ions reacting with, for example, carbon dioxide or carbonate ions to form calcium carbonate. They mostly consider how the chemical environment affects the formation of self-healing products and how to achieve agreement with experimental results [12,32,33,34,35,36,41,45,219].

However, these models have several limitations. First, they only simulate chemical reactions in solution and do not explicitly account for the change of the initial solid phase boundary due to the dissolution of soluble minerals at the fracture surface. Reaction diffusion models only include precipitation reactions in solution and do not simulate the dissolution reactions of the solid phase with a solution. Second, these models only uniformly simulate the healing process at the crack and do not accurately simulate the change in micro-morphology of the crack. The change in crack morphology is directly influenced by the concentration of aqueous substances and precipitations inside the solution [111]. In return, the change in crack morphology does affects the local concentrations of aqueous substances and precipitations in the solution. This interaction between the two factors is not reflected by existing models.

**Figure 7:**
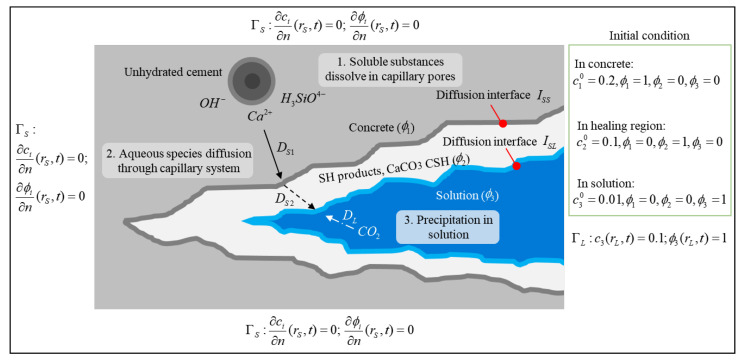
Schematic of the phase field model for the autogenous self-healing mechanism. $\Gamma_{S}$ and $\Gamma_{L}$ are the solid and liquid boundaries with coordinates $r_{S}$ and $r_{L}$, respectively; *n* is the outward unit normal vector.

A PF method can fill these gaps. Figure 7 shows schematically a potential application of a PF model for an autogenous self-healing mechanism. The solid–liquid phase distribution is described by an eigenfunction in the value range [0, 1]. The solid phase can be subdivided into an initial solid phase ($\phi_{1}$) and a healing solid phase ($\phi_{2}$), while $\phi_{3}$ represents the solution phase. The solid–solid ($I_{SS}$) and solid–liquid ($I_{SL}$) interfaces are simulated continuously. In addition to the solution ($D_{L}$), diffusion constants are distinguished between the concrete ($D_{S1}$) and the healing region ($D_{S2}$) due to differences in the meso- and microstructures. Neumann boundary conditions (Zero composition flux) were applied at the top, bottom, left and right (the light gray part) boundary for the solute concentration $c_{i}$ and the order parameter $\phi_{i}$. The Dirichlet boundary condition ($c_{3}$ = 0.1 and $\phi_{3}$ = 1) was applied at the right boundary (the blue part). The initial conditions are set based on the initial concentration in each phase. In this model, we chose to use the diffusion equation instead of Cahn–Hilliard equation because there is no phase separation. The Allen–Cahn equation is applied for solving the order parameter $\phi_{i}$.

This approach can accurately capture information about the alteration of the crack morphology due to solidification by the hydration reactions or the accumulation of precipitates [65,68,69].

With this, an overview of the PF approaches to the solute precipitation [180,220] and precipitation in binary alloys [179,221,222] is provided that are instructive for simulating self-healing mechanisms of concrete. The following models are presented in chronological order (Table 2).

### 5.1. Solute Precipitation

Solute precipitation is the process at which a solute changes from a liquid phase to a solid phase and precipitates outside its solution [230,231]. In fact, precipitates are mostly insoluble [232].

#### 5.1.1. Xu-Meakin Model, 2008

Xu and Meakin [180,220] developed a PF model for studying the dynamics of liquid–solid interfaces due to precipitation and/or dissolution of phases, based on the Karma–Rappel model [154] for pure melt solidifications. Discontinuities in the solute concentration at the interface are explicitly considered. An additional term has been added to the solute diffusion equation to describe the discontinuity of the solute concentration gradient at the interface. In addition, a detailed asymptotic analysis was used to establish a connection between the sharp interface and the PF model by correlating the reaction rate parameter k with the microscopic PF parameters. This ensures that the PF model will converge to the corresponding sharp-interface limit. A modified solute diffusion equation is built up as follows,

(4) $$\frac{\partial c}{\partial t}=D\nabla^{2}c+A_{1}\frac{\partial \phi }{\partial t}+A_{2}\frac{\partial \phi /\partial t}{\left|\nabla \phi \right|}\left(D\nabla^{2}\phi -\frac{\partial \phi }{\partial t}\right),$$

where the second additional term of the equation is corresponding to the discontinuity the solute concentration gradient at the interface. While the third additional term represents the net source or sink of the solute coming from the discontinuity in the solute concentration across the interface; *D* is the diffusion coefficient; $A_{1}$ and $A_{2}$ are two constants, which can be determined by the sharp-interface boundary conditions.

#### 5.1.2. Noorden-Eck Model, 2011

Van Noorden and Eck [179] proposed a PF model for a precipitation and/or dissolution process. The model describes a single-phase free boundary problem with dynamic conditions at the moving boundary. The concentration on the precipitate side of the interface is specified, and the velocity normal to the interface is nonlinear dependent to the concentration on the other side of the interface. The evolution equation of ϕ and *c* is described according to

(5) $$\frac{\partial \phi }{\partial t}=\frac{1}{\alpha }\Delta \phi -\frac{1}{\alpha \epsilon^{2}}p^{\prime }\left(\phi \right)-\frac{1}{\alpha \epsilon }\beta k^{\prime }\left(\phi \right)\left[f\left(c\right)+f^{\prime }\left(c\right)\left(c-\rho \right)\right];$$

(6) $$\frac{\partial c}{\partial t}=D\nabla \left[\nabla c+\left(\rho -c\right)\frac{k^{\prime }\left(\phi \right)}{k(\phi )}\nabla \phi \right],$$

where p(ϕ) is a double-well potential; f(c) is a rate function; k(ϕ) is an interpolation function; α, β, *D*, and ρ are physical parameters; and ϵ is the thickness of an interfacial layer.

Redeker and Rohde [221,222] extended the Noorden–Eck model by incorporating curvature effects between two fluid phases to simulate precipitation in a porous medium. The model contains two immiscible fluids and one solid phase. Dissolved ions in one of the fluids can precipitate at the pore boundaries. Bringedal et al. [226] considered not only the diffusion of ions in the fluid phase, but also the effect of fluid flow on precipitation.

### 5.2. Metal Precipitation

Unlike solute precipitation, metal precipitation occurs in a supersaturated solid solution. Metals and metal oxides exist in the form of crystals. A crystal is a structure in which its atoms or molecules are arranged in an orderly fashion according to certain rules. A crystal is pure when all the components are just a single substance or a compound. If there is another substance involved that occupies the original atomic location and does not destroy the original structure, then this is a solid solution [233]. The original component is equivalent to a solvent and the foreign component is equivalent to a solute. As with a solution, when the solute in a solid solution is supersaturated in the solvent, it can no longer remain stable in the crystal structure and eventually precipitates [234].

The precipitate particles are generally metallic compounds, but may also be formed by aggregation of solute atoms in supersaturated solid solutions in a number of small solute-rich regions [235]. The precipitated particles act as barriers to dislocation movement, allowing significant increase in strength and hardness of most structural alloys of aluminum, magnesium, nickel, and titanium, as well as some steels and stainless steels [236]. The precipitation mechanisms of different binary and ternary alloys have been intensively studied by using PF models [182,237,238].

#### 5.2.1. Wang–Chen Model, 1993

In the earlier study by Wang et al. [178], a PF model based on a microscopic kinetic model and elastic strain theory was developed to study the morphological evolution of the solid-state precipitation, controlled by transformation-induced elastic strain. The free energy of an inhomogeneous solid solution is given by the following equation,

(7) $$F\left(c\right)=\frac{1}{2}\sum_{\phi^{\prime }}W\left(r-r^{\prime }\right)c\left(r\right)c\left(r^{\prime }\right)+k_{B}T\sum_{r}\left[c\left(r\right)\ln c\left(r\right)+\left(1-c(r)\right)\ln\left(1-c\left(r\right)\right)\right]$$

where ϕ(r,t) is the non-equilibrium single crystal sites of solute atoms, *r* is the crystal lattice site, $W(r-r^{\prime })$ is the pairwise interaction energy of two atoms at the lattice site *r* and $r^{\prime }$, and $k_{B}$ is the Boltzmann’s constant. The drawback of this model is that the matrix phase and the precipitates are iso-structurally treated. However, this assumption does not apply to the simulation of Al-Li alloy precipitation.

#### 5.2.2. Rubin–Khachaturyan Model, 1999

Rubin and Khachaturyan [168] developed a 3D stochastic PF model for simulating the microstructural evolution of Ni-Al superalloys. This model considers the coherency strain in an elastic anisotropic system. The coarse grained stress-free free energy was expressed as

(8) $$\begin{array}{cc} F= & \int_{V}\left[\frac{1}{2}\left(\alpha_{ij}\nabla_{ic}\nabla_{jc}+\sum_{p=1}^{3}\beta_{ij}\left(p\right)\nabla_{i\phi_{p}}\nabla_{j\phi_{p}}\right)+f\left(c,\phi_{1},\phi_{2},\phi_{3}\right)\right]d^{3}r \end{array}$$

where $\alpha_{ij}$ and $\beta_{ij}\left(p\right)$ are the gradient coefficients, $\nabla_{ic}$ and $\nabla_{jc}$ denote the gradient terms of multi-composition profile c(r,t), $\nabla_{i\phi_{p}}$ and $\nabla_{j\phi_{p}}$ are the gradient terms of multi-component long-range order parameter ϕ(r,t), the specific free energy $f(c,\phi_{1},\phi_{2},\phi_{3})$ is approximated by a polynomial, and the second integral term is the total strain energy functional based on the Fourier transform microelasticity method.

#### 5.2.3. Chen–Ma Model, 2004

Chen et al. [177] designed a quantitative PF modeling scheme for multicomponent diffusion-controlled precipitate growth and dissolution in Ti-Al-V system in which the thermodynamic and kinetic data of existing databases CALPHAD was directly inserted into the PF model. The total Gibbs free energy is described as follows,

(9) $$G\left(T,c,\phi \right)=\frac{1}{V_{m}}\int_{V}\left[G_{m}\left(T,c_{i},\phi \right)+\sum_{i=1}^{n-1}\frac{k_{i}}{2}|\nabla c_{i}{|}^{2}+\frac{k_{\phi }}{2}{\left|\nabla \phi \right|}^{2}\right]dV.$$

where $G_{m}$ is the local molar Gibbs free energy; $k_{i}$ and $k_{j}$ are the gradient-energy coefficients for concentration and order parameter inhomogeneities, respectively; $V_{m}$ is molar volume.

The temporal evolution of the composition is governed by Cahn–Hilliard diffusion equation on the basis of the phenomenological Fick–Onsager equations

(10) $$\frac{1}{V_{m}^{2}}\frac{\partial c_{k}}{\partial t}=\nabla \sum_{j=1}^{n-1}M_{kj}\left(T,c_{i},\phi \right)\nabla \frac{\delta G}{\delta c_{i}}$$

where $M_{kj}$ are chemical mobilities related to atomic mobilities.

## 6. Phase-Field Modeling for Fracture Mechanisms

Fracture mechanics of concrete is a topic of intensive research during the last years. Simulation technology for analyzing crack initiation and propagation in concrete are numerous [58,239,240,241,242,243,244,245,246,247,248,249,250,251,252,253,254,255]. Besides boundary and finite element methods for linear elastic fracture analysis, different versions of the so called eXtended Finite Element Method (XFEM) are frequently applied [256,257].

Starting with the works of Bourdin et al. [258] and Miehe et al. [141], fracture processes were modeled explicitly by a PF approach. Due to its simplicity this methodology gained a wide interest and started to be used in the engineering community since 2010. From there on many scientist have worked in this field and developed PF approaches for finite elements methods (FEM), isogeometirc analysis (IGA), and recently also for the virtual element methods (VEM). The main driving force for these developments is the possibility to handle complex fracture phenomena within numerical methods in various dimensions. Thus, research on PF approaches is still actual and points in many different directions.

**Figure 8:**
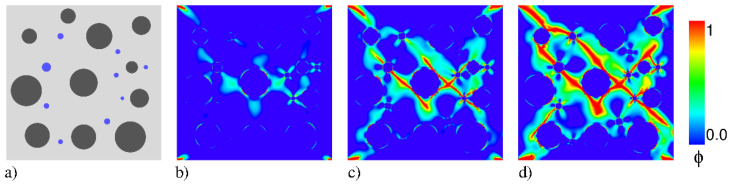
Concrete failure in poro-elasto-plastic media. (**a**) Schematic of the concrete idealized microstructure: Light gray color refers to the hydrated cement paste, dark gray color stands for the unhydrated clinker particles, and blue color depicts the water content. (**b**–**d**) Evolution of crack phase-field ϕ for different deformation states up to final failure, as outlined in [150].

In this review article, the simulation of fracture processes in concrete is achieved by utilizing the continuum PF method, which is based on the regularization of sharp crack discontinuities. This avoids the use of complex discretization methods for crack discontinuities and can account for multi-branched cracks within a solid skeleton (e.g., hydrated cement paste, unhydrated clinker particles, and stones). In particular due to the over-complicated geometry and content of concrete at multi-scales, in Figure 8 an example for PF modeling of water-induced failure mechanics in concrete microstructure is presented. In recent years, several brittle [259,260,261,262,263,264,265,266,267,268,269,270,271,272,273,274,275,276,277,278,279,280,281,282,283,284,285,286,287,288,289,290,291,292,293,294,295,296] and ductile [149,297,298,299,300,301,302,303,304,305,306,307,308,309,310,311,312,313,314,315,316,317,318,319,320,321,322,323,324] PF fracture formulations have been proposed in literature. These studies range from modeling 2D/3D small and large strain deformations, variational formulations, multi-scale/physics problems, mathematical analysis, different decompositions and discretization techniques with many applications in science and engineering. All these examples demonstrate the potential of PF method for crack propagation.

The aforementioned PF approaches consider the fracture behavior of concrete, i.e., as a crack initiation and propagation. However, an important aspect in concrete is the treatment of the crack-closure effects. This response was firstly investigated in the works [325,326] for fatigue crack closure under cyclic tension. Thereby, the results indicate a fatigue crack, propagating under zero-to-tension loading may be partially or completely closed at zero load. A review of this physical phenomena can be seen in [327,328,329]. To the author’s best knowledge, a PF approach for modeling crack closure is still an open issue. To this end, cohesive elements along the crack path will be coupled with the PF formulations to prevent overlapping of the crack faces. Another future direction is to use a contact scheme at the crack faces similar to the work developed by [330]. A further important aspect is the PF modeling of crack-closure induced by a self-healing mechanism (introduced in Section 3) in cementitious systems. These topics await investigation.

### 6.1. Fundamental Variational Formulations

In Griffith-type fracture formulations, the mechanical deformation denoted generally by “state” and the sharp crack surface Γ in a brittle elastic solid (e.g., cement paste) are determined by the incremental minimization problem developed by Francfort and Marigo [331] as

(11) $$E\left(state,\Gamma \right)=\int_{V\backslash \Gamma }f\left(state\right)\;dV+G_{c}\;H\left(\Gamma \right)\to \mathrm{Min}!$$

where $G_{c}$ is the Griffith critical surface energy release and H(Γ) is the Hausdorff surface measure of the crack set Γ. In Equation (11), the functional *E* has a structure identical to that for image segmentation developed by Mumford and Shah [332]. It consists of the strain energy stored in the solid as well as the energy release due to fracture.

### 6.2. Regularized Variational Theory

The numerical evaluation of the sharp crack interface in the functional *E* (Equation (11)) is not suitable within a standard finite element framework, as outlined in the work of Bourdin et al. [258]. Therefore, a regularized crack interface using a specific regularization profile γ is introduced in the studies of Miehe et al. [141,297]. It is based on a geometric regularization of sharp crack discontinuities that is governed by a crack PF

(12) $$\phi \in \left[0,1\right]\;\mathrm{with}\;\dot{\phi }\geq 0$$

It characterizes locally for the initial condition ϕ=0 the unbroken and for ϕ=1 the fully broken state of the material. Thus, the critical fracture energy is approximated by

(13) $$G_{c}\;H\left(\Gamma \right)\approx \int_{V}G_{c}\;\gamma \left(\phi ,\nabla \phi \right)\;dV\;\mathrm{with}\;\gamma \left(\phi ,\nabla \phi \right):=\frac{1}{2\;l_{f}}\;\phi^{2}+\frac{l_{f}}{2}\;{\left|\nabla \phi \right|}^{2}$$

in terms of the crack surface density function per unit volume of the solid. The regularization is governed by a fracture length scale $l_{f}$. Note that the limit for vanishing the fracture length scale $l_{f}\to 0$ gives the sharp crack surface Γ.

Therefore, the minimization problem represented by Equation (11) can be expressed in the following form,

(14) $$\tilde{E}\left(state,\Gamma \right)=\int_{V}\hat{W}\left(state,\phi ,\nabla \phi \right)\;dV\to \mathrm{Min}!$$

defined in terms of the total work density function $\hat{W}$ as

(15) $$\hat{W}\left(state,\phi ,\nabla \phi \right)=g\left(\phi \right)\;f\left(state\right)+G_{c}\;\gamma \left(\phi ,\nabla \phi \right)\;,$$

contains a degraded elastic work density and the crack energy release per unit volume. g(ϕ) is a degradation function defined as $g\left(\phi \right)={\left(1-\phi \right)}^{2}$. It describes the degradation of the solid with the evolving crack phase-field ϕ, as depicted in Figure 8b–d.

## 7. Discussion and Conclusions

Based on the above literature review, it can be observed that PF methods have a great potential for simulating self-healing mechanisms in concrete. Therefore, it can be applied to solve problems that cannot be addressed by commonly applied models. It has the potential of an unprecedented breakthrough. As self-healing of concrete is a rather complex process, it is an interaction between physical, chemical and mechanical mechanisms. Obtaining a novel, versatile model for self-healing concrete is a multidisciplinary study involving civil engineering, materials science, and chemistry. Many studies have been conducted in these fields using the PF approach, while it will be a great reference for the development of a self-healing PF model.

In future research, it would be recommended to include in the polynomial system of the PF approach the pore structure, concrete matrix, water dissolution, and hydration product phases at the crack front. In this way, the free-energy equations will combine hydration kinetics, crystallization kinetics, polymerization reaction kinetics, mass transport and chemical energies to provide a detailed description of the phase nucleation and growth mechanisms at the crack front. Coupling a reactive PF model with a fracture PF model allows to simulate the crack development and its mechanical self-healing recovery effects at different stages and under different environmental conditions. In order to achieve this goal, there are several self-healing mechanisms that need to be studied in great detail. Validating these models should be continuously done by comparing them with experimental results. The following potential future steps are identified:

1. Evolution of the pore structure at the crack surface:
  During the process of autonomous self-healing, soluble substances at the crack surface enter the solution and undergo various dissolution reactions, followed by hydration and carbonation crystallization reactions. Part of the solution will diffuse into the capillary pores of the concrete matrix, where crystallization and precipitation also occur. The growth of the cracked surface also forms a new pore structure, which further affects the diffusion and chemical reaction processes. Thus, the pore structure of the crack boundary is constantly changing with ongoing reaction. Its interaction with the crack morphology, reactant concentration, and mass transport needs to be investigated in the future.
2. Influencing factors and simulations for mechanical repair of cracks:
  The fracture PF part is a combination of elastic and fracture energies. Elastic free energy will follow the classical assumptions while the fracture part will account for the fracture toughness, order formulation, evolution equations, and healing regain laws. Moreover, both are closely related to the packing density field. This is because the mechanical properties at fracture mainly depend on the solid-phase continuity. The mechanical properties are enhanced in a homogeneously dense position of the filler and, conversely, worse in the disconnected parts of the solid phase. The packing density field, in turn, is related to the mass transport. Therefore, a numerical transport–mechanical coupling strategy shall be developed to simulate the overall performance of the self-healing mechanism.
3. Evolution of crack healing morphology:
  The morphology of the crack greatly influences its local healing effect. At the crack tip, healing products are produced faster and more frequently because of the higher concentration of reactants. The movement of the crack tip is faster than at other locations. Thus the crack morphology changes continuously with the healing process. As the PF model avoids tracking the boundary conditions at the interface and instead simulates the evolution of the auxiliary field. Therefore, the evolution of the interfacial morphology is easier to simulate. In addition, the simulation of interfacial morphology will take into account the distribution of bacteria, adhesive agents and mineral admixtures. Therefore, the macroscopic representation of a crack healing morphology shall be simulated from a micro-level point of view.
4. Free energy to distinguish between various product phases:
  Self-healing products contain multiple substances (CSH, CH, or additional byproducts) that, although they have the same healing mechanism (aggregation, crystallization and precipitation), their chemical reaction kinetics are different. This affects the rate of healing of the cracks as a whole. Therefore, the free energies of the various product phases and the corresponding thermodynamic parameters will be distinguished in the future and reflected in specific simulations.
5. Determination of PF parameters:
  A formulation for the determination of the PF parameters needs to be provided. Information on the PF parameters and their interrelationships will be obtained from thermodynamic and diffusion databases in combination with experimental data. Combined with the second law of thermodynamics and non-equilibrium thermodynamics, the self-diffusion, mutual diffusion, and chemical diffusion coefficients will be related to the diffusion mobility (*M*). The order parameter mobility (*L*) will be derived and their relationship to other phase-field parameters will be investigated.
6. Development of a three-dimensional model:
  As a self-healing process includes complex physical-chemical-mechanical processes, these mechanisms can only be accurately simulated in a fully three-dimensional system. Therefore, a three-dimensional simulation of the self-healing process need to be performed with realistic boundary conditions. The simulation results need to be verified and compared with 3D computed tomography scan (CT scan) results of concrete specimens.

In conclusion, the use of a PF method is feasible and has a significant application advantages in the field of self-healing concrete applications. Although this method still has a long way to go before it becomes a fully fledged simulation tool, these early studies are considered to be an important step towards reaching this goal.

## Figures and Tables

**Table 1:** Expressions, graphs, and applications of the local free energy.

Double-well		
$f\left(\phi \right)=A\left(-\frac{1}{2}\phi^{2}+\frac{1}{4}\phi^{4}\right);\phi \in \left(-1,1\right)$ where *A* is the height of the potential energy between the two states at the minimum free energy.		
solidification	[54,55,147,154,156,172,173,174,175,176,177,178,179,180]	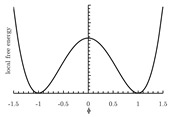
coarsening and grain growth	[127,128,181,182,183]	
dislocation dynamics	[184,185]	
crack propagation	[136,137,138,139,140]	
crystal growth under stress	[186,187]	
biological application	[188,189]	
phase transformations in thin films	[190]	
electrochemical process	[191,192,193,194,195]	
Double-obstacle		
$f\left(\phi \right)=\psi \left(\phi \right)+I_{\left[-1,1\right]}\left(\phi \right),$ where, $\psi \left(\phi \right)=A\left(1-\phi^{2}\right);$ $I_{\left[-1,1\right]}\left(\phi \right)=\left\{\begin{array}{cc} \infty & \;|\phi |>1\\ 0 & \;|\phi |\leq 1 \end{array}\right..$ when the phase transition only occurs in the narrow interface layer ϕ∈(−1,1) instead of in regions outside the interfacial layer.		
solidification	[196,197]	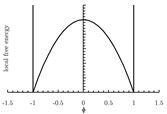
cell dynamical system	[198,199]	
stiffness maximization	[170]	
electromigration	[200,201]	
Crystalline energy		
$f\left(\phi \right)=A\sin^{2}\left(\pi \phi \right);\phi \in \left(-\infty ,+\infty \right),$ where *A* is the energy barrier between two neighboring minima. This function is formulated with an infinite number of degenerated minima.		
dislocation system	[158,159,202,203]	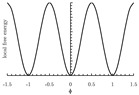
spiral growth	[160,204]	
Potential with temperature field		
$f\left(\phi ,T\right)=\frac{1}{8\alpha }{\left(1-\phi^{2}\right)}^{2}-\left(T_{i}-T_{m}\right)\phi$, where $T_{i}-T_{m}$ is the difference between the current temperature and the melting temperature; α is a positive constant.		
solidification	[161,162,163,164,165]	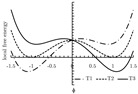
Landau-polynomial		
$f\left(\phi \right)=f_{dis}+A\phi +B\phi^{2}+C\phi^{3}+D\phi^{4}+E\phi^{5}+F\phi^{6}$, where $f_{dis}$ is the free energy of the disordered phase; A∼F are expansion coefficients related to temperature.		
solidification	[168,205,206,207,208]	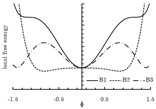
solid-state phase transformations	[166,167,209,210,211]	
electrochemical process	[169,212]	
crystal growth under stress	[170]	
phase transformations in thin films	[171,213,214]	

**Table 2:** PF models for precipitation mechanisms.

**Main** **Application**	**PF Model**	**Reference**	**Feature**
Solute precipitation	Xu-Meakin model	[180,220,223,224,225]	Discontinuity of the solute concentration gradient at the interface.
	Noorden-Eck model	[179,221,222,226]	a Single-phase free boundary problem with dynamic conditions at the moving boundary.
Metal precipitation	Wang-Chen model	[178,227]	Solid-state precipitation controlled by transformation-induced elastic strains.
	Rubin-Khachaturyan model	[168,228]	3D stochastic PF model.
	Chen-Ma model	[177,229]	Kinetic data of existing databases CALPHAD applied into the PF model.
